# Sexual dimorphism in the lasting effects of moderate caloric restriction during gestation on energy homeostasis in rats is related with fetal programming of insulin and leptin resistance

**DOI:** 10.1186/1743-7075-7-69

**Published:** 2010-08-26

**Authors:** Mariona Palou, Teresa Priego, Juana Sánchez, Andreu Palou, Catalina Picó

**Affiliations:** 1 Laboratory of Molecular Biology, Nutrition and Biotechnology (Nutrigenomics), University of the Balearic Islands (UIB) and CIBER Fisiopatología de la Obesidad y Nutrición (CIBEROBN), Palma de Mallorca, Spain

## Abstract

**Aim:**

We aimed to characterize the lasting effect of moderate caloric restriction during early pregnancy on offspring energy homeostasis, by focusing on the effects on food intake and body weight as well as on the insulin and leptin systems.

**Methods:**

Male and female offspring of 20% caloric restricted dams (from 1 to 12 days of pregnancy) (CR) and from control dams were studied. These animals were fed after weaning with a normal-fat (NF) diet until the age of 4 months, and then moved to a high-fat (HF) diet. Blood parameters were measured under fed and 14-h fasting conditions at different ages (2, 4 and 5 months). Food preferences were also assessed in adult animals.

**Results:**

Accumulated caloric intake from weaning to the age of 5 months was higher in CR animals compared with their controls, and this resulted in higher body weight in adulthood in males, but not in females. Both male and female CR animals already showed higher insulin levels at the age of 2 months, under fed conditions, and higher HOMA-IR from the age of 4 months, compared with their controls. CR male animals, but not females, displayed higher preference for fat-rich food than their controls in adulthood and higher circulating leptin levels when they were under HF diet.

**Conclusion:**

It is suggested that hyperinsulinemia may play a role in the etiology of hyperphagia in the offspring of caloric restricted animals during gestation, with different outcomes on body weight depending on the gender, which could be associated with different programming effects on later leptin resistance.

## Introduction

There is a growing body of evidence showing that the nutritional environment during early life may have later effects on the propensity to suffer from obesity and its related metabolic pathologies [1,2]. In this sense, maternal prenatal malnutrition has been described to have long term consequences on offspring metabolic energy regulatory systems, and has been associated with obesity in adult rats and humans [3,4]. The Dutch Hunger winter represents an emblematic example of this association. A higher incidence of obesity was found in men whose mothers underwent malnutrition during the first 2 trimesters of gestation due to the acute famine that ravaged the western part of Holland during the last 6 months of World War II [4]. Increasing epidemiological evidence also links low birth weight to an increased risk of developing adult diseases, including cardiovascular disease, hypertension, type 2 diabetes, and central obesity [5-7].

However, most evidence showing a direct association between gestational malnutrition and a higher propensity to obesity in later life comes from studies in rats, based on severe caloric restriction, generally between 30 to 50%, or severe protein restriction. For instance, Jones and Friedman [3] observed that male rats delivered from dams that were 50% caloric restricted during the first 2 weeks of pregnancy became obese after 5 weeks of age; however, the female offspring did not overeat and did not become obese. Results of Anguita et al [8] showed that a similar treatment during gestation in dams led to different effects in their offspring, which were influenced by gender. In males, the intrauterine malnutrition resulted in an impairment of normal weight gain and fat deposition from 1 to 53 days of age, in spite of normal food intake; in contrast, in females, this treatment led to a marked fat accumulation by 53 days of age, with normal food intake. Vickers at al [9], by focusing on male rats, also described clinical alterations in the offspring of 30% caloric restricted dams throughout pregnancy. These animals displayed hyperphagia, hypertension and greater fat accumulation (although without showing higher body weight) compared with their controls, and these alterations were further increased with advanced age and were amplified by hypercaloric nutrition. Most of the studies in this sense have addressed the effects of severe caloric restriction, which is known to result in marked fetal growth retardation, whereas, to the best of our knowledge, the lasting effects of moderate, less severe caloric restriction during gestation on energy homeostasis in both male and female offspring have not been directly studied. We consider this grade of restriction better fits within the normal range of human intake, and hence can be more relevant to human diet health.

On another hand, little is known about the underlying mechanisms involved in the developmental programming of energy balance under conditions of undernourishment during gestation. The involvement of specific areas of the hypothalamus in the regulation of food intake and energy homeostasis has been clearly established [10]. Hence, an impaired capacity to regulate energy homeostasis in adult life may be explained, at least in part, by a permanent "misprogramming" of key hypothalamic areas during critical periods of development, resulting in permanent dysfunctions in the neural control of metabolism, and thus affecting its capacity to integrate and process peripheral metabolic information [11]. In this sense, we have previously described that a moderate caloric restriction of 20% during the first 12 days of pregnancy affects the normal development of hypothalamic structure and function, particularly factors involved in insulin and leptin central action, and impairs hypothalamic response to fed/fasting conditions in weaned rats, suggesting a predisposition to central insulin and leptin resistance [12]. However, the lasting effects of this treatment during development were not addressed in that study.

The purpose of the present study was to examine the lasting effects of moderate caloric restriction of 20% during the first 12 days of gestation upon offspring energy homeostasis, by focusing on the effects on food intake, body weight and fat accumulation, and on the insulin and leptin systems as possible determinants of potential disorders. In addition, the study was designed to investigate differences between male and female rats and in the effects of a dietary stressor in adult life such as high fat (HF) diet exposure.

## Materials and methods

### Animals and experimental design

The study was performed in male and female rats from 12 different litters, following the protocol below. All rats were housed under controlled temperature (22°C) and a 12 h light-dark cycle (light on from 0800 to 2000), and had unlimited access to tap water and standard chow diet (3 kcal/g, with 2.9% calories from fat; Panlab, Barcelona, Spain) unless mentioned otherwise. Briefly, virgin female Wistar rats weighing between 200 g and 225 g were mated with male rats (Charles River Laboratories, Barcelona, Spain). Day of conception (day 0 of pregnancy) was determined by examination of vaginal smears for the presence of sperm, and then female rats were single caged. Pregnant rats were divided into two groups (6 animals/group): one with free access to standard chow diet, and the other one underwent a 20% restriction of caloric intake from day 1 to day 12 of pregnancy. Caloric restriction was performed by offering each dam a daily amount of food corresponding to 80% of the calories should be eaten according to body weight. This amount was calculated considering the calories daily consumed by their control animals under *ad libitum *feeding conditions. After the caloric restriction period, rats were allowed to eat *ad libitum*, and food intake was measured. At day 1 after delivery, excess pups in each litter were removed to keep 10 pups per dam (five males and five females, when possible). Weaning was conducted at 21 days of life.

At weaning, 24 animals from control dams (controls) (12 males and 12 females) and 24 from caloric restricted dams (CR) (12 males and 12 females) were placed two per cage, paired with another animal of the same group, and fed with standard diet until the age of 4 months; then they were exposed to a high fat (HF) diet (4.7 kcal/g, with 45% calories from fat, Research Diets, Inc., NJ, USA). HF diet contained 5.5% calories from soybean oil and 39.5% from lard.

Body weight and food intake of the offspring were followed until the age of 5 months. In addition, body fat content (by EchoMRI-700™, Echo Medical Systems, LLC., TX, USA) was measured without anesthesia at 2 different ages, when animals were 2 and 5 months old. Body length (from the tip of the nose to the anus) was also measured at the end of the follow up, when animals were 5 months old.

At 3 different ages (2, 4 and 5 months), blood samples (n = 8 animals/group) were collected (in heparinized containers) from the saphenous vein of control and CR animals, under different feeding conditions: *ad libitum *- animals provided with free access to chow diet - and fasting - animals deprived of food for 14 h. In both conditions, samples were obtained without anesthesia and during the first 2 h of the beginning of the light cycle. Plasma was obtained by centrifugation of blood at 700 g for 10 min, and stored at -20°C until analysis.

The animal protocol followed in this study was reviewed and approved by the Bioethical Committee of our University and guidelines for the use and care of laboratory animals of the University were followed.

### Measurement of circulating parameters under fed/fasting conditions, and calculation of the homeostatic model assessment for insulin resistance at different ages

Blood glucose concentration was measured by Accu-Chek Glucometer (Roche Diagnostics, Barcelona, Spain). Plasma insulin concentration was determined using a rat insulin enzyme-linked immunosorbent assay (ELISA) kit (Mercodia AB, Uppsala, Sweden) following standard procedures. Plasma leptin concentration was measured using a mouse leptin ELISA kit (R&D Systems, Minneapolis, MN). Commercial enzymatic colorimetric kits were used for the determination of plasma triglyceride (TG) levels (Triglyceride (INT) 20, Sigma Diagnostics, St Louis, MO, USA) and non-esterified fatty acid (NEFA) (Wako Chemicals GmbH, Neuss, Germany).

The homeostatic model assessment for insulin resistance (HOMA-IR) was used to assess insulin resistance. It was calculated from fasting insulin and glucose concentration using the formula of Matthews et al [13]: HOMA-IR = fasting glucose (mmol/liter) × fasting insulin (mU/liter)/22.5.

### Two-bottle preference test

Food preferences were assessed by a two-bottle preference test as previously described [14]. Briefly, the rats had to choose between two bottles containing either a carbohydrate (CHO)-rich liquid diet or a fat-rich liquid diet. The two diets had identical caloric density (2.31 kcal/g) and the following ingredients: for the CHO-rich diet, 10 g/100 ml skimmed milk, 40 g/100 ml sucrose, 4 g/100 ml olive oil, and 0.35 g/100 ml xanthan gum (Sigma, Madrid, Spain); and for the fat-rich diet, 10 g/100 ml skimmed milk, 10 g/100 ml sucrose, 17.3 g/100 ml olive oil, and 0.35 g/100 ml xanthan gum. Before the test started, and over a period of 8 d, the rats were habituated to each bottle given individually on alternate days for 1 h, without withdrawing the standard chow diet. The test was started 2 d after the adaptation period. Solid food was withdrawn at the beginning of the light phase. Two bottles containing pre-weighed quantities of either the CHO- or fat-rich diet were placed side-by-side 4 h after the beginning of the light cycle for 1 h. The bottles were then reweighed, and the intake of each diet was determined and corrected for spillage. Spillage was estimated by weighing small collection plates placed underneath the spout of the bottles. The test was performed in adult animals (n = 8), when rats were 3 months old.

### Statistical analysis

Given that the animals studied were from six different litters in each treatment group, the effect of litter was simultaneously factored with all data by repeated measures ANOVA. No interactions between the litter and treatment were found across all the data, thus, data were expressed as mean ± S.E.M of animals from the six different litters. Multiple comparisons were assessed by repeated measures ANOVA and two-way ANOVA to determine the effects of different factors (age, sex, caloric restriction during pregnancy and feeding conditions). Single comparisons between groups were assessed by Student's t test or Paired t test. The analyses were performed with SPSS for Windows (SPSS, Chicago, IL). P < 0.05 was always the threshold of significance.

## Results

### Body weight gain and cumulative food intake of dams

As previously described in the same cohort of dams [12], 20% maternal food restriction during the first period of gestation resulted in lower weight gain, and, consequently, body weight of caloric restricted dams at the end of the restriction period (day 13) was significantly lower than that of their controls (258 ± 9 g and 238 ± 4 g, respectively) (Student's t test). However, at the end of the pregnancy, no significant differences were found between body weights of both groups of animals: 323 ± 4 g for control dams and 325 ± 12 g for caloric restricted dams. No significant differences were found in body weight gain or cumulative food intake of dams during lactation (data not shown).

### Body weight and food intake in the offspring

**Figure 1:**
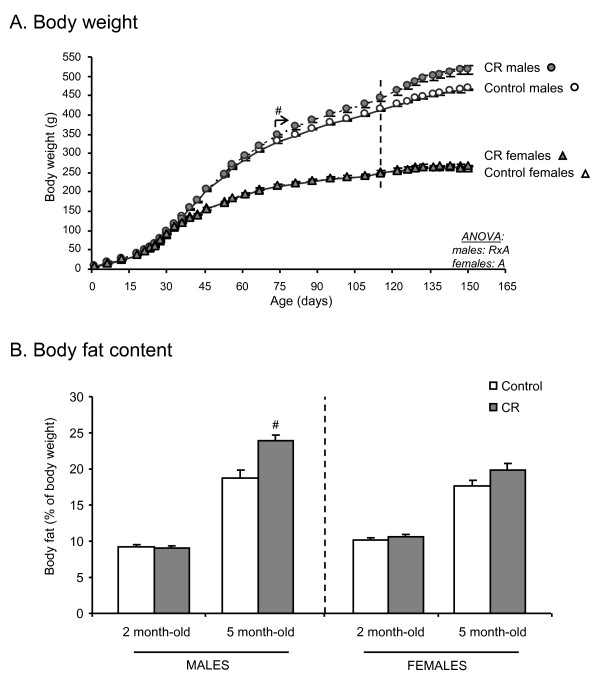
**Body weight over time until the age of 5 months (A) and body fat content at the ages of 2 and 5 months (B) of male and female offspring of controls and caloric restricted dams during gestation (CR)**. Animals were fed with standard normal fat (NF) diet until the age of 4 months and then exposed to a high fat (HF) diet (the dotted line indicates the time point of change from NF to HF diet). Data are expressed as the mean ± SEM of 12 animals per group. Statistics: *A*, effect of age; *RxA*, interaction between caloric restriction during lactation and age (ANOVA repeated measures). ^#^, different from their respective control group (Student's t test). The arrow indicates the starting point of significant effects on body weight in male animals.

As shown in Figure 1A, moderate maternal caloric restriction during the first 12 days of pregnancy did not result in different offspring body weight at birth with respect to their controls. However, in adulthood, CR male animals gained more weight and displayed higher body weight than their controls from day 74 of life onwards (Student's t test). When animals were 4 months old (just before changing to HF diet), CR male animals weighed 6.9% more than their controls, and the difference was even higher (11%) when animals were 5 months old and were under HF diet. Body weight gain of animals during the HF diet period (from 4 to 5 months old) was also significantly higher in CR male animals (21 ± 1%) compared with their controls (17 ± 1%).

**Figure 2:**
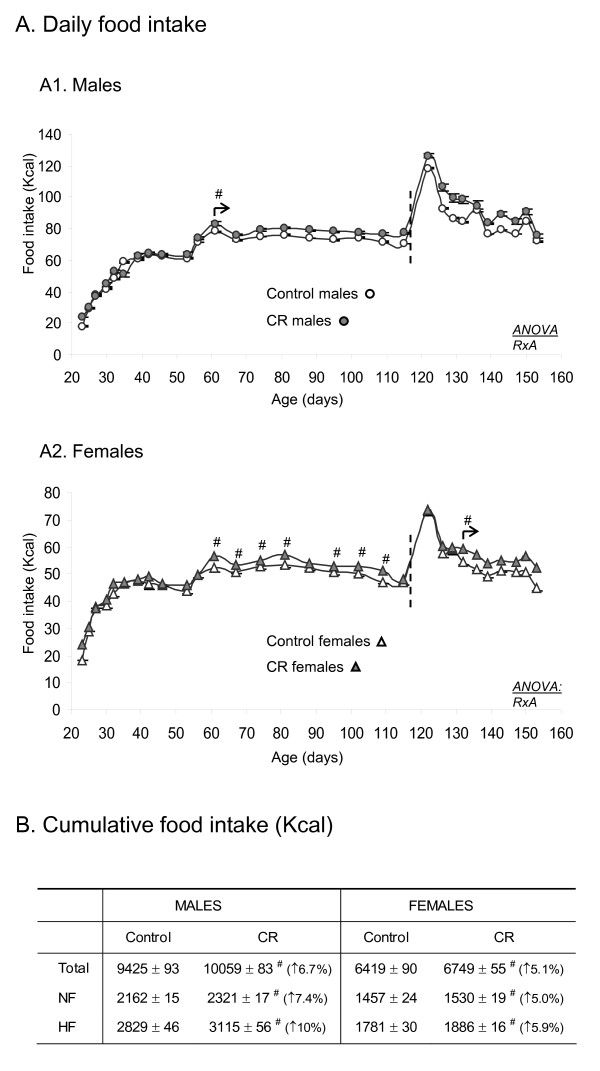
**Daily food intake (A1, in males and A2, in females) and cumulative caloric intake (B) from weaning at the age of 21 days until the age of 5 months (TOTAL), as well as during the last month under NF diet (from 3 to 4 months old) (NF) and when animals were exposed to HF diet (from 4 to 5 months old) (HF) of male and female offspring of controls and caloric restricted dams during gestation (CR)**. The percentage increase in food intake of CR compared with controls is indicated in brackets. Animals were fed with standard normal fat (NF) diet until the age of 4 months and then exposed to a high fat (HF) diet (the dotted line indicates the time point of change from NF to HF diet). Data are expressed as the mean ± SEM of 12 animals per group. Statistics: *RxA*, interaction between caloric restriction during lactation and age (ANOVA repeated measures). ^#^, different from their respective control group (Student's t test). The arrow indicates the starting point of significant effects on body weight in male animals.

The higher body weight of CR male animals compared with their controls can be explained by higher food intake (Figure 2A). Daily food intake of CR male animals was higher than controls from day 56 of life onwards. Accumulated caloric intake of animals from weaning at day 21 until the age of 5 months was significantly higher than their controls (increase of 6.7%), the differences were greater when animals were exposed to HF diet (from 4 to 5 months old; increase of 10%) compared with the last month under NF diet (from 3 to 4 months old; increase of 7.4%) (Figure 2B).

Unlike males, no statistically significant differences were found in body weight of CR female animals compared with their controls at any age (Figure 1A). Nevertheless, CR females gained slighlty more weight than their controls during the period of HF diet feeding (8.1 ± 0.9% in controls and 10 ± 1% in CR) (Student's t test). Interestingly, CR female animals also ate more calories than their controls from day 56 onwards (with the exception of a couple of days) (Student's t test) (Figure 2A). Accumulated caloric intake from weaning until the age of 5 months was also significantly higher than controls (increase of 5.1%) (Student's t test), with a higher increase during the period under HF diet (increase of 5.9%) compared with the last month under NF diet (increase of 5.0%) (Figure 2B).

Differences in body weight between control and CR adult male animals can be mainly attributed to differences in body fat content. At the age of 5 months, CR male animals presented higher percentage of body fat than controls (Student's t test) (Figure 1B). However, in accordance with body weight, percentage of body fat was not significantly different between control and CR female animals. At the age of 2 months, both control and CR male and female animals showed no differences in their body fat content.

Body length of animals was not different between control and CR animals, either in males or females (data not shown).

### Circulating parameters under fed and fasting conditions in the offspring

Circulating glucose, insulin, leptin, triglycerides and NEFA levels were measured in control and CR male and female animals under fed and 14 h fasting conditions at the ages of 2, 4 and 5 months (Table 1).

**Table 1:** Blood parameters

		Glucose (mg/dl)		Insulin (μg/l)		Leptin (μg/l)		Triglycerides (mg/ml)		NEFA (μg/l)	
		Control	CR	Control	CR	Control	CR	Control	CR	Control	CR

MALES											
	Ad libitum	98 ± 4	95 ± 4	0.774 ± 0.166	1.44 ± 0.27^#^	3.52 ± 0.43	3.38 ± 0.44	0.669 ± 0.193	1.48 ± 0.20^#^	0.447 ± 0.054	0.895 ± 0.054^#^
2 month-old	14-h fasting	65 ± 2^+^	66 ± 3^+^	0.115 ± 0.029^+^	0.103 ± 0.026^+^	0.832 ± 0.053^+^	0.471 ± 0.086^+^	0.447 ± 0.065	1.30 ± 0.24^#^	0.622 ± 0.077	1.21 ± 0.03^+ #^
	*ANOVA*	*F*		*RxF*		*F*		*R*		*R,F*	

	Ad libitum	73 ± 2	77 ± 2	1.44 ± 0.24	2.29 ± 0.60	7.34 ± 0.74	9.48 ± 0.71	1.25 ± 0.25	1.57 ± 0.13	0.765 ± 0.079	1.12 ± 0.16
4 month-old	14-h fasting	64 ± 3^+^	72 ± 3	0.468 ± 0.094^+^	1.15 ± 0.29^+ #^	4.23 ± 0.31^+^	4.82 ± 0.33^+^	0.636 ± 0.153^+^	1.12 ± 0.10^+ #^	0.842 ± 0.144	1.35 ± 0.11^#^
	*ANOVA*	*F*		*F*		*F*		*R,F*		*R*	

	Ad libitum	77 ± 5	80 ± 2	1.47 ± 0.41	2.69 ± 0.68^#^	8.17 ± 1.25	15.3 ± 1.6^#^	1.06 ± 0.16	1.94 ± 0.23^#^	1.27 ± 0.07	1.17 ± 0.14
5 month-old	14-h fasting	63 ± 3^+^	65 ± 3^+^	0.583 ± 0.147^+^	1.43 ± 0.35^+ #^	4.44 ± 0.78^+^	6.17 ± 0.84^+^	1.02 ± 0.21	1.31 ± 0.21^+^	1.20 ± 0.10	1.26 ± 0.07
	*ANOVA*	*F*		*R,F*		*RxF*		*R*			

FEMALES											
	Ad libitum	92 ± 3	100 ± 5	0.657 ± 0.115	1.55 ±0.35^#^	3.38 ± 0.44	2.87 ± 0.32	0.373 ± 0.063	0.946 ± 0.142^#^	0.529 ± 0.057	0.921 ± 0.047^#^
2 month-old	14-h fasting	71 ± 2^+^	71 ± 3^+^	0.134 ± 0.019^+^	0.134 ±0.014^+^	0.448 ± 0.055^+^	0.511 ± 0.129^+^	0.516 ± 0.148	0.633 ± 0.217	0.657 ± 0.085	1.28 ± 0.11^+ #^
	*ANOVA*	*F*		*RxF*		*F*		*R*		*R,F*	

	Ad libitum	84 ± 6	82 ± 2	1.27 ± 0.16	1.86 ±0.35	5.59 ± 0.26	6.31 ± 0.37	0.747 ± 0.176	1.66 ± 0.38^#^	0.715 ± 0.090	1.35 ± 0.15^#^
4 month-old	14-h fasting	67 ± 3^+^	67 ± 1^+^	0.246 ± 0.045^+^	0.465 ±0.120^+^	2.03 ± 0.17^+^	2.09 ± 0.10^+^	0.496 ± 0.119^+^	0.693 ± 0.102^+^	0.826 ± 0.105	1.26 ± 0.08^#^
	*ANOVA*	*F*		*F*		*F*		*R,F*		*R*	

	Ad libitum	80 ± 1	81 ± 3	1.47 ± 0.19	2.34 ± 0.33^#^	7.21 ± 0.67	6.21 ± 0.449	0.909 ± 0.290	1.44 ± 0.30	0.957 ± 0.068	1.10 ± 0.09
5 month-old	14-h fasting	69 ± 2^+^	60 ± 3^+ #^	0.251 ± 0.033^+^	0.476 ± 0.103^+^	2.98 ± 0.20^+^	3.25 ± 0.162^+^	1.14 ± 0.32	1.19 ± 0.29	1.17 ± 0.09	1.30 ± 0.12
	*ANOVA*	*F*		*R, F*		*F*					

Circulating glucose, insulin, leptin, triacylglicerides and non-esterified fatty acid (NEFA) in male and female offspring of controls and caloric restricted dams during gestation (CR) at the ages of 2, 4 and 5 months, under ad libitum feeding conditions and after 14 h fasting conditions. Animals were fed with standard normal fat (NF) diet until the age of 4 months and then exposed to a high fat (HF) diet. Data are mean ± S.E.M. (n = 8). Statistics: *R*, effect of caloric restriction; *F *effect of fasting; *RxF*, interaction between caloric restriction and fasting (ANOVA repeated measures). ^#^, different from their respective control group (Student's t test); ^+^, different from fed conditions (Paired t test).

No significant differences were found in glucose levels between control and CR animals in either male or female animals, although 5-month-old female CR animals, under fasting conditions, showed lower glucose levels than their controls (Table 1). However, both male and female CR animals at the age of 2 months under feeding conditions showed higher insulin levels compared with their respective controls (RxF interaction) (Table 1). The tendency to higher insulin levels was maintained under both fed and fasting conditions when animals were 4 months old (although differences only reached statistical significance for CR males at the age of 4 months under fasting conditions, Student's t test), but were further increased when animals were 5 months old (two way ANOVA).

**Figure 3:**
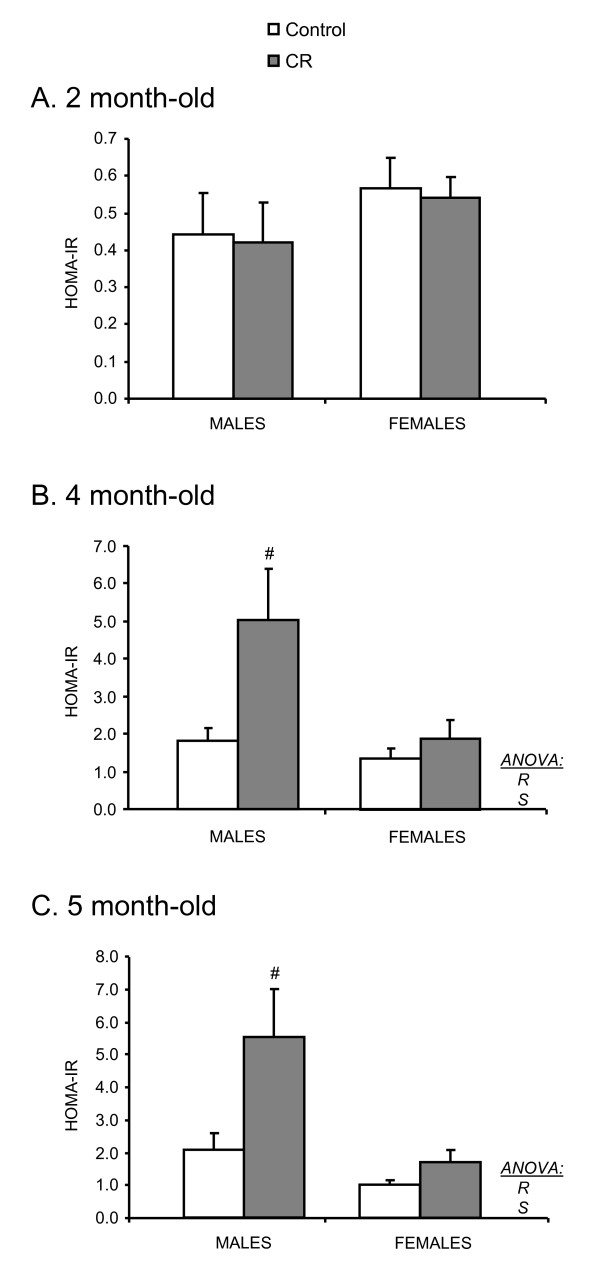
**HOMA-IR index of male and female offspring of controls and caloric restricted dams during gestation (CR) at the ages of 2, 4 and 5 months**. Animals were fed with standard normal fat (NF) diet until the age of 4 months and then exposed to a high fat (HF) diet. Data are mean ± S.E.M. (n = 8). Statistics: *R*, effect of caloric restriction; *S*, effect of sex (two way ANOVA). ^#^, different from their respective control group (Student's t test).

The HOMA-IR index was also calculated to estimate insulin sensitivity. This value was not different between control and CR animals at the age of 2 months, but was significantly higher in CR male and female animals at the ages of 4 and 5 months (two way ANOVA) (Figure 3).

Circulating leptin levels of control and CR animals under fed and fasting conditions at the ages of 2, 4 and 5 months are also shown in Table 1. No significant differences were found concerning leptin levels at the ages of 2 and 4 months in male animals, although at the age of 5 months, CR male animals displayed higher leptin levels under fed conditions (Student's t test) than their controls, with no significant differences under fasting conditions. Leptin levels in female animals were not different between control and CR animals either under fed or fasting conditions at any of the ages studied.

Blood lipid profile was significantly altered in CR animals as of the early stages of life (Table 1). In fact, CR male and female animals already displayed higher circulating TG levels than their controls at the age of 2 months, and differences were maintained at the ages of 4 and 5 months in males, but only at the age of 4 months in females. Circulating NEFA levels were also significantly higher in male and female CR animals compared with their controls at the ages of 2 and 4 months, but not at the age of 5 months.

### Food preferences in the offspring

**Figure 4:**
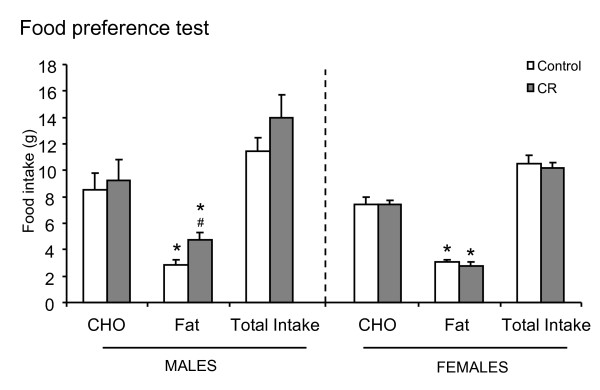
**Dietary preferences measured by the two-bottle preference test of male and female offspring of controls and caloric restricted dams during gestation (CR) at the age of 3 months**. Bars represent the mean ± S.E.M of the amount of carbohydrate (CHO) rich food, fat rich food (Fat) and total (Cho + Fat) food ingested in the choice test of 8 animals per group. The two diets had identical caloric density (2.31 kcal/g). Statistics: ^#^, different from their respective control (Student's t test); *, Fat *vs *CHO diet (Paired t test).

Food preferences were measured in adult animals at the age of 3 months with the two-bottle preference test (Figure 4). Both control and CR male and female rats showed a lower preference for fat-rich food in comparison with CHO-rich food (Paired t test); even so, the preference for fat-rich food was higher in CR male rats compared with their controls (Student's t test). No significant effects were found between controls and CR female animals concerning food preferences.

## Discussion

It is known that severe caloric restriction during gestation in rats increases the propensity of adverse health outcomes in adulthood, including obesity, although differences according to the magnitude of restriction has been described [3,8,9]. Here we show that a more moderate caloric restriction (of 20%) during the first part of gestation also has lasting effects in the offspring, programming animals for higher food intake. In male animals, this results in higher body weight gain and higher fat accumulation in adulthood. However, female animals seem to be protected against the accumulation of excess fat, at least when they are under NF diet, and partially later on, when animals were 5 months old and exposed to HF diet. Another study made by exposing pregnant rats to a 50% caloric restriction during the first 2 weeks of gestation also showed that the male offspring became hyperphagic and gained more weight than the controls [3], while the female progeny did not overeat and did not become obese. The different outcome of this study compared with the present study lies basically in the effects on food intake in female rats. However the causes of the differences between both studies, regardless the different magnitude of restriction, are not known.

Central resistance to insulin and/or leptin has been proposed as important mechanisms responsible for the dysregulation of energy homeostasis, which may lead to obesity [15-17]. Here we show that the male and female offspring of dams that underwent moderate caloric restriction during gestation displayed higher circulating insulin levels than their controls; this occurred at a juvenile age, and previous to any apparent effect on body weight. Although increased plasma insulin levels should be associated with reduction in appetite [18], the hyperinsulinemia seen in CR animals, associated with hyperphagia, is likely to reflect reduced central insulin action. Nevertheless, no significant differences in insulin levels were found under fasting conditions between control and CR animals, and no differences were found either in the HOMA-IR index at this age. However, a higher HOMA-IR value was found later on in life, when animals were 4 and 5 months old. These results seem to confirm that moderate caloric restriction during fetal life may program for an early onset of insulin resistance, which may lead to hyperphagia and this may in turn be responsible, although only in male animals, for the later onset of obesity.

Concerning leptin, moderate caloric restriction led to gender related differences on later leptinemia. Adult CR male animals under NF diet showed a tendency to higher circulating leptin levels under ad libitum feeding conditions compared with their controls, in accordance with their higher body weight. This hyperleptinemia was further increased later on, when animals were 5 months old and under HF diet. In fact, it has been described an increase in leptin levels with age, which seems to be associated to the increased body weigh and fat content [19]. This may contribute to the dysregulation of energy balance that occurs with aging, which may be responsible for the age-associated proneness to obesity [20]. Unlike males, CR females did not display significant changes in circulating leptin levels compared with their controls. Thus, on the one hand, considering the central effects of leptin inhibiting food intake, the hyperleptinemia seen in CR male animals would suggest that leptin is not able to fully exert its inhibitory effects on appetite which would be indicative of a loss of leptin sensitivity. On the other hand, the fact that CR female rats maintain leptinemia at control levels and are protected against an excess of fat accumulation compared with their controls, may suggest that leptin is able to exert its effects on body weight control, even though they eat more calories. It could be speculated that leptin may be more effective increasing energy expenditure to compensate its higher food intake, but this issue has not been explored in this study. All in all, these results suggest that fetal programming of certain leptin resistance and the resulting hyperleptinemia, in the male offspring of caloric restricted animals, may be critical in the development of obesity. However, it should be stressed that since we have not directly measured leptin sensitivity in this study, we can not confirm the existence of leptin resistance but only hyperleptinemia. On the other hand, it must be also pointed out that the increased leptinemia occurring in CR male animals at the age of 5 months compared with controls could also be reflecting the difference in adiposity, but considering that leptin resistance is today recognized as a significant contributor to obesity [21], the excess of fat accumulation would appear to be more a consequence rather a cause of the hyperleptinemia.

Impaired fetal growth by 30% caloric restriction throughout gestation has also been described to be associated with hyperinsulinemia and hyperleptinemia in adult male offspring [9]. These disorders were also proposed to be responsible for the hyperphagia and obesity developed in these animals. It must be highlighted that the restriction used in this previous article by other authors [9] resulted in fetal growth retardation, reflected by significantly lower body weight of the pups at birth, this model resembling the human conditions of children born with intrauterine growth retardation [22]. In our model, with less restrictive conditions, body weight at birth was not significantly different between control and CR animals, but the lasting effects, at least in males, seem to be comparable to those described in the study mentioned.

Circulating lipid profile in animals that underwent caloric restriction during gestation, particularly the elevations of plasma NEFA and TG levels already occurring at the age of 2 months, could also give some clues to explain the apparent insulin resistance in both male and female CR animals. Elevated circulating NEFA levels have been described to play an important role in early molecular events involved in the development of insulin resistance [23]. They directly affect insulin signalling, diminish glucose uptake in muscle, drive exaggerated triglyceride synthesis and induce gluconeogenesis in the liver [24]. In addition, insulin-resistant adipocytes are characterized by low liposynthetic capacity and high lipolytic capacity, causing increased release of fatty acids [25]. On the other hand, results of Zammit [26] have demonstrated that repeated exposure of the liver to elevated levels of insulin has a potent stimulatory effect on hepatic TG production. Thus, this over-stimulation of hepatic TG production through insulin action may outline a mechanistic basis for the development of leptin resistance, even independently of HF diet feeding [27]. Elevated fasting plasma TG levels have also been described in the offspring of 30% caloric restricted dams throughout pregnancy [27], and this has been found in conjunction with hyperinsulinemia and leptin resistance. Here, an elevation in TG and NEFA levels was found in both male and female CR animals, also in conjunction with an apparent insulin resistance, while hyperleptinemia and overweight features were only found in adult male animals.

Metabolic programming of hyperphagia due to maternal caloric restriction during gestation could be associated with an alteration in the neuronal development of the central nervous system under these environmental conditions [12]. In fact, it has been previously described that animals that underwent caloric restriction during gestation, in the same conditions as in the present study, exhibited after weaning an alteration in the number of neurons in the ARC nucleus, with a reduction in the number of NPY+ and of αMSH+ neurons [12]. This may alter the normal function of this hypothalamic area, making it insufficiently robust to maintain energy homeostasis in adult life. In addition, caloric restricted animals showed decreased expression levels of insulin receptor, and higher expression of SOCS-3 after weaning, suggesting that these animals were programmed for a lower sensitivity to insulin [12]. The results of our current study may further confirm the pre-existence of insulin resistance as a consequence of fetal programming under these conditions. Moreover, CR animals also showed diminished mRNA expression levels of the long form of the leptin receptor at weaning [12] suggesting a predisposition to leptin resistance. Notably, this effect was found in both male and female animals; however, in the present study, hyperleptinemia seems to occur only in CR adult males, with no apparent sings in females. This does not preclude the possible of development of hyperleptinemia or leptin resistance in female animals later on in life or when coupled to other environmental or dietary stressors. These results may suggest that the final effects of caloric restriction during pregnancy may be the product of secondary responses to the programming challenge that in turn promote adaptation that manifest later in life, and in a sex-dependent manner.

The reasons for the differences between males and females regarding circulating leptin levels and fat accumulation in adult life due to these particular prenatal conditions are not clearly elucidated yet. Of interest, differences between genders concerning the sensitivity to central leptin and insulin have been previously described [28,29]. The brain of female rats, compared with that of males, has been described to be relatively more sensitive to the action of leptin, while males seem to be more sensitive to insulin [28]. Differences in gonadal hormones can account, at least in part, for these differences, since estrogen acts within the brain to increase leptin sensitivity and decrease insulin sensitivity [29]. Notably, other studies based on postnatal dietary manipulations, such as cafeteria diet exposure [30] or HF diet exposure [31] have shown that male rats, but not females, exhibited decreased hypothalamic expression levels of leptin receptor compared with their controls under standard diet, suggesting central leptin resistance. Moreover, decreased expression of leptin receptor has also been observed in internal white adipose tissue depots of male rats exposed to HF diet, but not in females [32]. Thus, according to these considerations, male rats may appear to be more prone to suffer central and/or peripheral leptin resistance under certain prenatal or postnatal manipulations, and this could help to explain the greater tendency of males to suffer from obesity-linked disorders under certain environmental conditions. According to the results of Clegg et al [29], it could be hypothesised that programming of changes in the endocrine milieu, particularly sex steroid hormones, as a consequence of caloric restriction during gestation might contribute to explain the apparent gender-related differences concerning leptin sensitivity and body weight gain; however further analyses need to be done to bear out this point.

In addition to the amount of food intake, programming food preferences, as a part of the feeding behavior, may contribute to body weight control and to the development of obesity. This is particularly relevant in humans where obesity development is generally associated with increased appetite and preference for highly caloric food [33,34]. Here, we show that moderate caloric restriction during gestation also affected food preferences of adult male offspring. Although both control and CR male and female animals had a preference for CHO-rich food compared with fat-rich food, the preference for fat-rich food was higher in CR males compared with their controls. Considering that CR animals seem to be programmed for greater food intake, the concomitant preference of CR male animals for HF food may contribute to explain why these animals eat more calories than their controls when exposed to HF diet, and why the difference was even higher than observed under NF diet. This higher preference for fat-rich food was not observed in CR female animals compared with their controls. Other studies related with modifications of perinatal nutrition have also described changes in food preferences in later life. For example, moderate caloric restriction during lactation in dams, which has been associated with a certain protection against body weight gain in adulthood, resulted in different effects on food preferences in the offspring [35]. These animals, particularly females, showed less preference for fat-rich food when exposed to a HF diet compared with their unrestricted controls [35]. In addition, oral supplementation with leptin during lactation protects animals against diet induced obesity [14,36] and enhances preferences for CHO-rich foods rather than fat-rich foods [14]. On the other hand, maternal protein restriction during fetal life is associated with an obesogenic phenotype in adulthood and causes an increase of fat food preferences [37]. Thus, nutritional changes during critical stages of development, may program the control of feeding behaviour mechanisms, affecting both appetite and food preferences, being important in the susceptibility to suffer obesity.

In summary, it is concluded that a moderate caloric restriction of 20% during the first part of gestation has lasting, gender-dependent effects in the offspring. In particular, these animals are programmed for higher food intake, which seems to be related with central insulin resistance and with higher blood TG and NEFA levels, already present at a juvenile age, and this concludes in higher body weight in adult male rats but not in females. Why programming animals for higher food intake has different outcomes depending on the gender of animals needs further investigation; it is hypothesised that hyperleptinemia, which seems to occur, at least to some extent, in CR males but not in females under these conditions, may play a determinant role in the adult onset of obesity.

## Competing interests

The authors declare that they have no competing interests.

## Authors' contributions

MP participated in the experimental design of the study, carried out the animal procedure and the analysis, and participated in the discussion of the results. TP participated in the animal procedure and the discussion of the results. JS participated in the animal procedure and in the discussion of the results. AP participated in the design and coordination of the study and revised the final version of the manuscript. And CP conceived of the study, and participated in its design and coordination, carried out the discussion of the results and wrote the article. All authors read and approved the final manuscript.
